# Effects of phosphodiesterase 3A modulation on murine cerebral microhemorrhages

**DOI:** 10.1186/s12974-017-0885-7

**Published:** 2017-06-05

**Authors:** Rachita K. Sumbria, Vitaly Vasilevko, Mher Mahoney Grigoryan, Annlia Paganini-Hill, Ronald Kim, David H. Cribbs, Mark J. Fisher

**Affiliations:** 1 Department of Biopharmaceutical Sciences, School of Pharmacy, Keck Graduate Institute, Claremont, CA USA; 2 Department of Neurology, University of California, Irvine, CA USA; 3 Institute for Memory Impairments and Neurological Disorders, University of California, Irvine, CA USA; 4 Department of Pathology & Laboratory Medicine, University of California, Irvine, CA USA; 5 Department of Anatomy & Neurobiology, University of California, Irvine, CA USA; 6 UC Irvine Medical Center, 101 The City Drive South, Shanbrom Hall, Room 121, Orange, CA 92868 USA

**Keywords:** Phosphodiesterase 3A, Cerebral microhemorrhage, Cerebral microbleeds, Cilostazol, Cerebral amyloid angiopathy

## Abstract

**Background:**

Cerebral microbleeds (CMB) are MRI-demonstrable cerebral microhemorrhages (CMH) which commonly coexist with ischemic stroke. This creates a challenging therapeutic milieu, and a strategy that simultaneously protects the vessel wall and provides anti-thrombotic activity is an attractive potential approach. Phosphodiesterase 3A (PDE3A) inhibition is known to provide cerebral vessel wall protection combined with anti-thrombotic effects. As an initial step in the development of a therapy that simultaneously treats CMB and ischemic stroke, we hypothesized that inhibition of the PDE3A pathway is protective against CMH development.

**Methods:**

The effect of PDE3A pathway inhibition was studied in the inflammation-induced and cerebral amyloid angiopathy (CAA)-associated mouse models of CMH. The PDE3A pathway was modulated using two approaches: genetic deletion of PDE3A and pharmacological inhibition of PDE3A by cilostazol. The effects of PDE3A pathway modulation on H&E- and Prussian blue (PB)-positive CMH development, BBB function (IgG, claudin-5, and fibrinogen), and neuroinflammation (ICAM-1, Iba-1, and GFAP) were investigated.

**Results:**

Robust development of CMH in the inflammation-induced and CAA-associated spontaneous mouse models was observed. Inflammation-induced CMH were associated with markers of BBB dysfunction and inflammation, and CAA-associated spontaneous CMH were associated primarily with markers of neuroinflammation. Genetic deletion of the PDE3A gene did not alter BBB function, microglial activation, or CMH development, but significantly reduced endothelial and astrocyte activation in the inflammation-induced CMH mouse model. In the CAA-associated CMH mouse model, PDE3A modulation via pharmacological inhibition by cilostazol did not alter BBB function, neuroinflammation, or CMH development.

**Conclusions:**

Modulation of the PDE3A pathway, either by genetic deletion or pharmacological inhibition, does not alter CMH development in an inflammation-induced or in a CAA-associated mouse model of CMH. The role of microglial activation and BBB injury in CMH development warrants further investigation.

## Background

Cerebral microbleeds (CMB) represent perivascular depositions of blood degradation products in the brain and are a common MRI finding in cerebrovascular disease, especially stroke and cerebral amyloid angiopathy (CAA) [1]. The prevalence of CMB ranges between 18 and 68% in patients with ischemic stroke, and presence of CMB is associated with an increased risk for development of intracerebral hemorrhage (ICH) [2]. Routine ischemic stroke prevention strategies require anti-thrombotic agents (platelet medications and anti-coagulants), which may increase the risk for hemorrhage in patients with CMB [2, 3]. Presence of CMB thus indicates a hemorrhage-prone state of the cerebral blood vessels, which may be exacerbated with anti-thrombotic agents. Coexisting ischemic and hemorrhagic processes (“mixed cerebrovascular disease”) thus pose a challenge to the stroke neurologist. A therapeutic strategy that simultaneously protects the vessel wall and has anti-thrombotic activity is therefore an attractive potential approach for mixed cerebrovascular disease.

Phosphodiesterases (PDEs) are enzymes that catalyze the hydrolysis of cyclic nucleotides (cyclic adenosine 3′-5′-monophosphate (cAMP) and cyclic guanosine 3′-5′-monophosphate (cGMP)) [4, 5]. Platelets possess several PDEs (PDE2, PDE3, PDE5) that modulate the level of cAMP and cGMP that are critical for platelet function [6]. PDE inhibitors interfere with platelet activation pathways by inhibiting the degradation of and thereby increasing the intracellular levels of cAMP and cGMP. Among the various PDEs, PDE3 has a high affinity for both cAMP and cGMP with a preference for cAMP, and the primary PDE3 isoform that regulates platelet function is PDE3A [7]. The PDE3A inhibitor cilostazol has been shown to be clinically effective for secondary prevention of ischemic stroke [8, 9]. In both in vitro and in vivo experiments, PDE3A inhibition appears to provide blood vessel wall protection by enhancing blood–brain barrier (BBB) properties [10–12] and reducing hemorrhagic injury [12–15]. These observations provide a strong rationale for utilizing PDE3A inhibition as therapy for brain microhemorrhagic disorders.

Given the above rationale, the aim of the current study was to examine the effect of PDE3A pathway inhibition on development of cerebral microhemorrhages (CMH), the pathological substrate of CMB. We studied CMH development in two well-characterized mouse models: the inflammation-induced mouse model of CMH [16] and an amyloid precursor protein transgenic (APP/Tg2576) mouse model that develops CAA with spontaneous CMH [17]. The overall hypothesis of the current study was that the inhibition of the PDE3A pathway is protective against inflammation-induced and CAA-associated CMH. To test this hypothesis, two approaches for PDE3A pathway modulation were used: genetic deletion of PDE3A and pharmacological inhibition of PDE3A by cilostazol. The effects of PDE3A pathway modulation on CMH development, BBB function, and neuroinflammation were therefore investigated.

## Methods

### Animals and treatment

All animal procedures followed the “Principles of Laboratory Animal Care” (NIH Publication No. 85-23), were approved by the University of California, Irvine, Institutional Animal Care and Use Committee, and followed the ARRIVE Guidelines for animal experiment reporting. To study the effects of PDE3A inhibition on CMH development, two approaches—genetic deletion and pharmacological inhibition of the PDE3A pathway—and two mouse models—the inflammation-induced mouse model of CMH and an amyloid precursor protein transgenic (APP/Tg2576) mouse model that develops CAA and spontaneous CMH—were used.

### Genetic deletion of PDE3A and CMH development

PDE3A knockout (KO) mice were used in the inflammation-induced mouse model of CMH. Heterozygous PDE3A^+/−^ KO breeding pairs (generously provided by Dr. Vincent Manganiello [7, 18]) were bred in-house to maintain a colony of homozygous PDE3A^−/−^ KO and wild-type (WT) littermate controls. All mice used in the study were 16–18 months, and the average weight of the WT and PDE3A^−/−^ KO mice was 44 ± 1.9 and 41 ± 1.5 g, respectively.

**Fig. 1:**
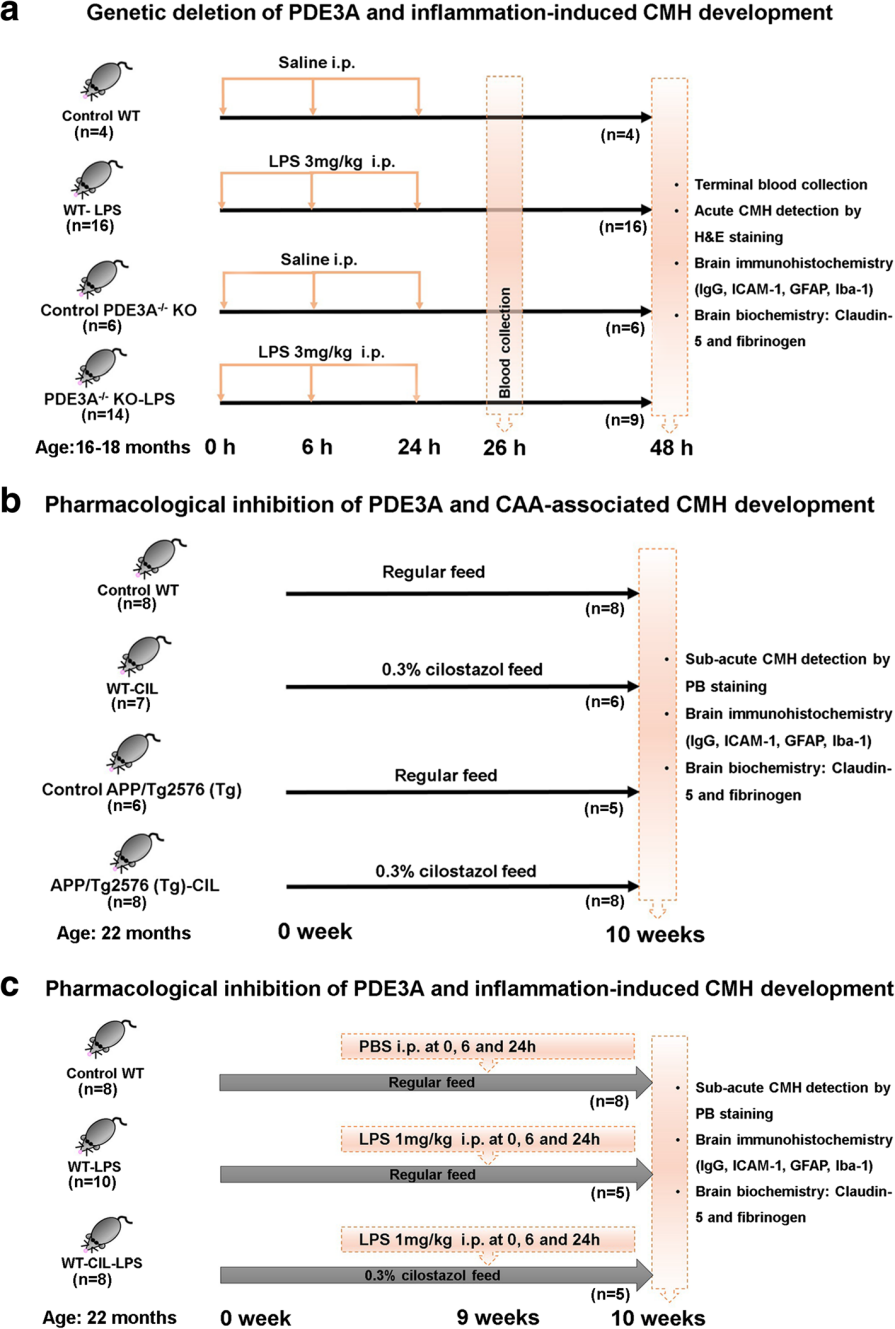
Experimental designs used in the current study. Workflow and treatment groups used to determine the effect of genetic deletion of PDE3A on inflammation-induced CMH development (**a**), pharmacological inhibition of PDE3A with cilostazol on CAA-associated spontaneous CMH development (**b**), and pharmacological inhibition of PDE3A with cilostazol on inflammation-induced CMH development (**c**)

Lipopolysaccharide (LPS) derived from the gram-negative bacterium *Salmonella typhimurium* (Sigma-Aldrich, St. Louis, MO) was used to induce CMH development, as described previously [16]. Briefly, PDE3A^−/−^ KO mice (*n* = 14) and WT littermate controls (*n* = 16) were given LPS (3 mg/kg) intraperitoneally (i.p.) at 0, 6, and 24 h. Control PDE3A^−/−^ KO mice (*n* = 6) and control WT littermates (*n* = 4) received an equal volume of sterile saline i.p. (Teknova, Hollister, CA). Mice fed and drank ad lib and received 1 mL sterile saline subcutaneously (s.q.) on an as-needed basis to prevent dehydration. Mice were monitored for 48 h, after which they were sacrificed (Fig. 1a). Mice that died prematurely were excluded from histochemical and biochemical analysis, but were used for survival analysis.

### Pharmacological inhibition of PDE3A and CMH development

The effect of pharmacological inhibition of PDE3A by cilostazol, a selective PDE3A inhibitor [19], on CMH development was studied in both the inflammation-induced and CAA-associated APP/Tg2576 (Tg) mouse models. To determine the achievable plasma cilostazol concentration, 4-month-old Tg mice and WT littermates were fed with mouse chow formulated with 0.03, 0.3, or 3% of cilostazol (Harlan, Madison, WI) (six WT and six Tg mice in each group), for 2 weeks. Plasma was collected before introduction of the cilostazol diet (T0) and after 2 weeks on the diet (T1). Cilostazol plasma levels were measured on HPLC by Sumika Analysis & Evaluation Service (Shanghai) Ltd.

To study the effect of cilostazol on CAA-associated CMH, 22-month-old Tg mice and WT littermates were housed under a 12-h light–dark cycle with free access to chow and water. Mice were divided into four groups: Tg mice fed regular chow (Tg; *n* = 6); Tg mice fed 0.3% cilostazol diet formulated in regular chow (Tg-CIL; *n* = 8); WT mice fed regular chow (WT; *n* = 8); and WT mice fed 0.3% cilostazol formulated in regular chow (WT-CIL; *n* = 7), for 10 weeks (Fig. 1b). Mice that died prematurely during the course of the 10 weeks were excluded from histochemical and biochemical analysis, but were used for survival analysis.

Nine weeks after initiation of the cilostazol or regular diet regimen, LPS derived from the gram-negative bacterium *S. typhimurium* was administered (1 mg/kg dose of i.p. at 0, 6, and 24 h) to a separate series of WT mice fed regular chow (WT-LPS; *n* = 10) and WT mice fed 0.3% cilostazol formulated in regular chow (WT-CIL-LPS; *n* = 8) to study the effect of pharmacological inhibition of the PDE3A pathway on inflammation-induced CMH development. WT mice fed regular chow and treated with an equal volume of PBS (i.p. at 0, 6, and 24 h) served as control mice (WT-PBS; *n* = 8). Mice were kept under standard vivarium conditions for 7 days after the first LPS or PBS injection and observed twice daily (Fig. 1c). Mice that died prematurely during the course of the 7 days were excluded from histochemical and biochemical analysis, but were used for survival analysis.

### Brain preparation

Forty-eight hours after the first LPS or saline injection (for genetic manipulation studies) or 10 weeks after initiation of cilostazol or regular diet treatment (for pharmacological inhibition studies), animals were anesthetized with a lethal dose of Nembutal (150 mg/kg, i.p.), cardiac perfusions were performed using ice-cold PBS for 5 min to flush the cerebral vasculature, and the brains were harvested immediately. The left cerebral hemispheres were flash-frozen using dry ice and stored at −80 °C for biochemical analyses. The right hemispheres were fixed in 5 mL of 4% paraformaldehyde (BM-154, Boston BioProducts, Ashland, MA) at 4 °C for 72 h for histological analyses.

### Cerebral microhemorrhage detection

Fixed mouse hemi-brains were examined for grossly visible surface CMH using a ×10 magnifying glass. Surface microhemorrhages were quantified and photographed using Canon PC2054 20 megapixel (Canon, Tokyo, Japan). Fixed brains were placed in an agarose mold and sectioned into 40-μm coronal sections with a vibratome (Technical Products International, Inc., St. Louis, MO). Every 4th, 5th, 6th, and 7th section was collected, and approximately 30 sections were analyzed per mouse brain.

The effect of PDE3A KO (genetic manipulation of the PDE3A pathway) on acute CMH development was assessed by examining the formation of hematoxylin and eosin (H&E)-positive CMH in a 2-day study, as described previously [20]. Prussian blue (PB) staining to detect CMH was not used for the PDE3A KO acute study based on previous studies from our lab [20]. For H&E staining, every 6th section (approximately 30 sections per mouse brain) was stained with H&E by the research service core at UCI Medical Center’s Department of Pathology and Laboratory Medicine. CMH were identified as a collection of red blood cells (RBC) that appear red-orange under H&E staining using a light microscope with a ×20 objective by an observer blinded to the experimental groups. All brain sections were examined, and all identified CMH were photographed using an Olympus BX40 microscope and CC-12 Soft-Imaging System with Olympus Microsuite™-B3SV software. Digitized images were analyzed using NIH ImageJ version 1.46r to determine CMH number, size (μm^2^), and positive area (sum of the area occupied by all the individual H&E-positive CMH expressed as a percentage of the total area analyzed) per mouse by an observer blinded to the experimental groups. To determine the total area analyzed, H&E-stained slides were scanned using the Canon MP250 scanner (Canon, Tokyo, Japan) under 600 dots per inch (DPI). The total area of each brain section was quantified using NIH ImageJ software and added to determine the total tissue area that was analyzed per mouse. The total CMH positive area was then normalized to the total tissue area for each mouse and expressed as a percentage of the total area analyzed, as mentioned above.

To study the effect of the PDE3A inhibitor cilostazol on CAA-associated CMH development, the PB method for hemosiderin (a marker of subacute CMH) was used [17, 20]. Briefly, three coronal sections per brain with coordinates −1.2, −2.2, and −3.2 mm posterior to the bregma selected according to *The Mouse Brain in Stereotaxic Coordinates* were stained with PB and counterstained with nuclear fast red [21]. All brain sections were examined, images of microscopic hemorrhages were taken using Olympus BX-UCB microscope with a ×20 objective, and CMH were analyzed using NIH ImageJ software by an investigator blinded to the experimental groups. Eclipse tool was used as a convex hull to encircle the CMH to determine the average size and total positive area of the PB-positive lesion in square micrometers based on the incorporated scale bar. For the total PB-positive area, individual PB-positive lesions for all the three brain sections were summed. The number, average size, and total area of PB-positive CMH per section were reported as described previously [17].

### TNF-α and β-TG ELISA

In the genetic manipulation studies, blood samples were obtained before and 26 and 48 h after LPS or saline injection via the retro-orbital sinus using heparinized capillary tubes (Fisher Scientific, Hampton, NH). For this, mice were briefly anesthetized using 3% isoflurane. Blood samples were collected on ice and centrifuged at 8000 × *g* for 15 min at 4 °C. For TNF-α (a marker of systemic inflammation) ELISA, supernatant was collected and assayed using a TNF-α ELISA kit (Life Technologies, Carlsbad, CA) as per the manufacturer’s protocol. For β-thromboglobulin (β-TG; a marker of platelet activation) ELISA, blood was collected in 1-mL Eppendorf tubes prefilled with a plasma collection solution (21% by volume of collected blood) to prevent in vitro platelet activation and assayed using a β-TG ELISA kit (US Biological, 023697, Salem, MA) as per the manufacturer’s instructions. The plasma collection solution for β-TG comprised acid–citrate–dextrose (ACD) National Institutes of Health formula A (Sigma-Aldrich, St. Louis, MO), aspirin (Sigma-Aldrich, St. Louis, MO), and prostaglandin E1 (Sigma-Aldrich, St. Louis, MO) at a 100:8:1 ratio, respectively [22].

### Immunohistochemistry

Immunohistochemistry was performed for ICAM-1 (inflammation-inducible protein intercellular adhesion molecule-1; a marker of endothelial cell activation), IgG (a marker of BBB damage), Iba-1 (ionized calcium-binding adaptor molecule 1; a marker of microglia/macrophages), and GFAP (glial fibrillary acidic protein; a marker of astrocytes). Immunohistochemical analyses [16] were performed using one coronal 40-μm section per mouse approximately −2 to −2.4 mm posterior to the bregma, and identical coordinate sections per staining from the analyzed groups were incubated in 0.5% hydrogen peroxide in 0.1 M PBS (pH 7.4) containing 0.3% Triton X-100 (PBST) for 30 min at room temperature to block endogenous peroxidase activity. After washing with PBST, sections were incubated for 30 min with PBST containing 2% bovine serum albumin (BSA) to block non-specific protein binding. Sections were then incubated overnight at 4 °C with a rabbit anti-mouse IgG antibody (1:200 dilution; Jackson ImmunoResearch, West Grove, PA), rabbit monoclonal antibody against ICAM-1 (1:500 dilution; Abcam, Cambridge, MA), rabbit antibody against Iba-1 (1:200 dilution; Wako Chemicals USA, Richmond, VA), or rabbit antibody against GFAP (1:2000 dilution; Abcam, Cambridge, MA). After washing with PBST, sections were incubated at room temperature for 1 h with biotinylated anti-rabbit IgG (1:500 dilution; Jackson ImmunoResearch, West Grove, PA), followed by 1 h incubation at room temperature with ABC complex according to manufacturer instructions (Vector Laboratories, Burlingame, CA). Sections were developed with 3,3′-diaminobenzidine (DAB) (Vector Laboratories, Burlingame, CA). Sixteen images per brain section from the frontal cortex, hippocampus, and thalamus areas were acquired randomly at ×20 magnification, and the total positive immunoreactive area (expressed as % of the total area analyzed) was quantified using NIH ImageJ software by an observer blinded to the experimental groups.

### Claudin-5 and fibrinogen western blot

Frozen left cerebral hemispheres were pulverized, the powder was homogenized in T-PER buffer (Thermo Fisher Scientific, Waltham, MA) with protease inhibitor cocktail (Roche Applied Science, Indianapolis, IN), and soluble fraction was collected after 100,000*g* centrifugation for 1 h at 4 °C. Protein concentrations for western blot analysis were determined using the Bradford protein assay, and approximately 50 μg of protein was resolved on SDS-PAGE 4–12% gel (Invitrogen, Carlsbad, CA). Primary antibodies for claudin-5 (tight junction protein) (Abcam, Cambridge, MA) and fibrinogen (a marker of BBB permeability) (US Biological, Salem, MA) were used at 1:2000 dilution followed by HRP-conjugated donkey anti-rabbit secondary antibody (Jackson Immuno Research, West Grove, PA). NIH ImageJ software was used to quantify western blot band intensities. Control protein GAPDH (Santa Cruz Biotechnology, Dallas, TX) was used to normalize band intensity measurements.

### Statistical analysis

Data were represented as mean ± SEM, and all statistical analyses were performed using GraphPad Prism 5 (GraphPad Software Inc., La Jolla, CA). To compare more than two groups, one-way ANOVA with Bonferroni’s post hoc test was used for normal data and the Kruskal-Wallis test with Dunn’s post hoc test for non-normal data. The Student *t* test was used to compare two groups with normally distributed data. Survival curves were analyzed using the Log-rank test. One-sample *t* test was used to compare group means with a hypothesized mean = 0 when the values in the control group were zero (e.g., surface microhemorrhages and TNF-α levels in the control mice). A *p* value of <0.05 was considered statistically significant.

## Results

### Survival

**Fig. 2:**
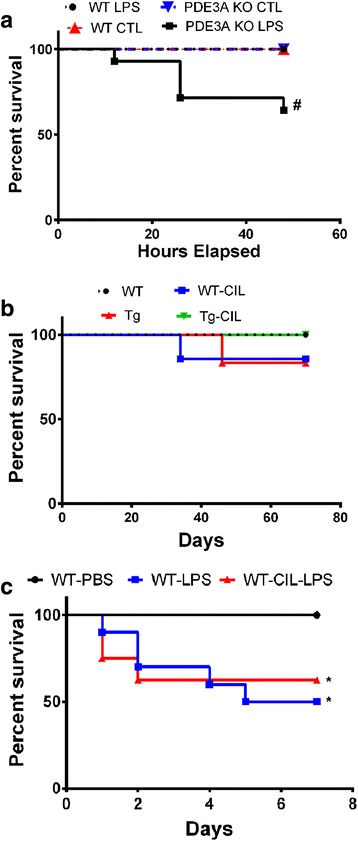
Survival analysis for the different experimental groups. Significant decrease in survival of PDE3A KO mice treated with LPS compared with the WT, PDE3A KO, and LPS-treated WT mice while studying the effect of genetic deletion of PDE3A on inflammation-induced CMH development (**a**). No difference in the survival between any experiment groups while studying the effect of pharmacological inhibition of PDE3A with cilostazol on CAA-associated CMH development (**b**). LPS treatment caused a significant reduction in survival in the WT and WT-CIL treated mice compared with PBS-treated WT mice while studying the effect of pharmacological inhibition of PDE3A with cilostazol on inflammation-induced CMH development (**c**). Statistical test: Log-rank test for survival analysis. #*p* < 0.05 compared with WT, PDE3A KO, and LPS-treated WT, *p < 0.05 compared with WT-PBS

While studying the effect of genetic deletion of PDE3A on CMH development, all the control mice (control PDE3A^−/−^ KO (*n* = 6) and control WT littermates (*n* = 4)) and LPS-treated WT mice (*n* = 16) survived the duration of the study. The LPS-treated PDE3A^−/−^ KO mice had a significantly lower (*p* < 0.01) survival (9 of 14) compared with the LPS-treated WT mice (Fig. 2a).

While studying the effect of pharmacological inhibition of PDE3A with cilostazol on CAA-associated CMH development, the survival of mice was as follows: 8/8 in the WT group, 6/7 in the WT-CIL group, 5/6 in the Tg group, and 8/8 in the Tg-CIL group (Fig. 2b). No significant difference in the survival was observed between any experiment groups. While studying the effect of pharmacological inhibition of PDE3A with cilostazol on inflammation-induced CMH development, the survival of mice was as follows: 8/8 in the WT-PBS group, 5/10 in the WT-LPS group, and 5/8 in the WT-CIL-LPS group (Fig. 2c). LPS treatment caused a significant reduction (*p* < 0.05) in survival in the WT and WT-CIL treated mice compared with the PBS-treated WT mice.

### Genetic deletion of PDE3A and surface cerebral microhemorrhages

**Fig. 3:**
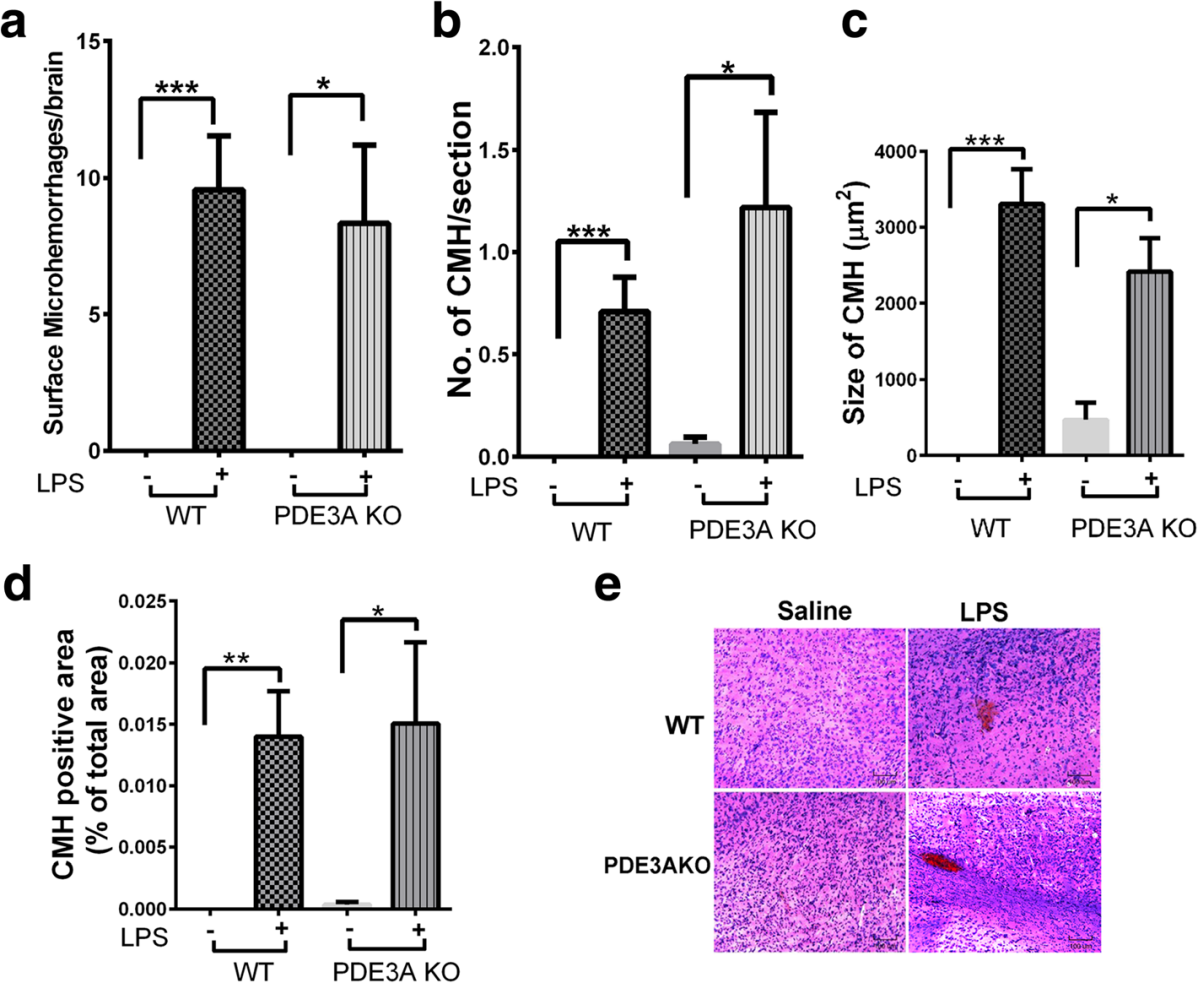
Genetic deletion of PDE3A and inflammation-induced CMH development: Significant increase in grossly visible surface CMH in the LPS-treated WT and LPS-treated PDE3A KO mice compared with their respective saline controls (**a**). Significant increase in H&E-positive parenchymal CMH number (**b**), size (**c**), and area (**d**) in the LPS-treated WT and LPS-treated PDE3A KO mice compared with their respective saline controls. Representative images of H&E-positive CMH in saline- and LPS-treated WT and PDE3A^−/−^ KO mice (**e**). Data are presented as mean ± SEM. Statistical test: one-way ANOVA with Bonferroni’s post-test or Kruskal-Wallis test with Dunn’s post-test for parenchymal CMH and one-sample *t* test with a hypothesized mean = 0 for surface CMH. *p < 0.05, **p < 0.01, ***p < 0.001

No grossly visible CMH were visible on the brain surface of mice treated with saline, in either WT or PDE3A^−/−^ KO mice. A 3 mg/kg dose of LPS at 0, 6, and 24 h induced the development of grossly visible surface microhemorrhages 48 h after the first LPS injection, in both WT (9.5 ± 1.9 per brain; *p* < 0.05) and PDE3A^−/−^ KO (8.3 ± 2.9 per brain; *p* < 0.05) mice compared with saline controls (Fig. 3a). Genetic deletion of PDE3A had no significant effect on LPS-induced surface microhemorrhage development, and no significant difference between LPS-treated WT and LPS-treated PDE3A^−/−^ KO mice was observed.

### Genetic deletion of PDE3A and H&E-positive acute parenchymal cerebral microhemorrhages

Negligible H&E-positive acute parenchymal CMH were observed in saline-treated WT (0.021 ± 0.008 per brain section) and PDE3A^−/−^ KO (0.06 ± 0.03 per brain section) mice. A 3 mg/kg dose of LPS at 0, 6, and 24 h significantly increased the formation of H&E-positive acute parenchymal CMH in both the WT (0.71 ± 0.16 per brain section) and PDE3A^−/−^ KO (1.2 ± 0.46 per brain section) mice compared with their respective saline-treated controls (Fig. 3b). There was a trend toward a higher H&E-positive CMH number in the LPS-treated PDE3A^−/−^ KO mice compared with the LPS-treated WT mice (*p* > 0.05).

LPS treatment produced H&E-positive CMH that were significantly larger in size compared with the saline controls, in both the WT and PDE3A^−/−^ KO mice. However, the mean CMH size did not differ significantly between LPS-treated WT and LPS-treated PDE3A^−/−^ KO mice (Fig. 3c). Similarly, the total CMH load (or the CMH positive area) was significantly higher in the LPS-treated WT and LPS-treated PDE3A^−/−^ KO mice compared with their respective saline-treated controls, but no difference between LPS-treated WT and LPS-treated PDE3A^−/−^ KO mice was observed (Fig. 3d). Representative images of H&E-positive CMH in the saline- and LPS-treated WT and PDE3A^−/−^ KO mice are shown in Fig. 3e. Overall, genetic deletion of PDE3A did not significantly alter H&E-positive inflammation-induced acute CMH development in the current study.

### Serum TNF-α and β-TG levels

**Fig. 4:**
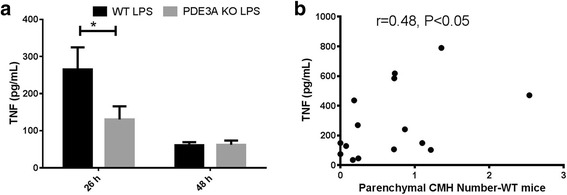
Significant increase in serum TNF-α levels in LPS-treated WT mice compared with LPS-treated PDE3A KO mice, 26 h after the first LPS injection (or 2 h after the last LPS injection) (**a**). No significant difference in the serum TNF-α levels between the LPS-treated WT mice compared with the LPS-treated PDE3A KO mice, 48 h after the first LPS injection (**a**). No detectable TNF-α levels were observed in the saline-treated WT and PDE3A KO mice. Significant correlation between the serum TNF-α levels of LPS-treated WT mice and number of H&E-positive CMH (*N* = 15). (**b**). Data are presented as mean ± SEM (**a**) and scatter plot with Spearman correlation coefficient (**b**). Statistical test: Student’s *t* test for TNF-α level comparison at 26 h and Spearman correlation coefficient. *p < 0.05

TNF-α levels were close to the detection limit in the saline-treated WT and PDE3A^−/−^ KO mice (data not shown). Two hours after the last LPS injection (or 26 h after the first LPS injection), serum TNF-α levels were significantly elevated in both the WT (265 ± 60 pg/mL) and PDE3A^−/−^ KO (131 ± 35 pg/mL) mice and the TNF-α levels were significantly higher in the WT mice compared with the PDE3A^−/−^ KO mice (Fig. 4a). TNF-α levels continued to be significantly elevated at 48 h after the first LPS injection compared with saline controls; however, no significant difference was observed between LPS-treated WT (61 ± 8 pg/mL) and LPS-treated PDE3A^−/−^ KO (62 ± 11 pg/mL) mice (Fig. 4a). Serum TNF-α levels were significantly associated (Spearman *r* = 0.48, *p* < 0.05) with the CMH load in the LPS-treated WT mice (Fig. 4b), but no significant correlation was observed in the LPS-treated PDE3A^−/−^ KO mice. No significant difference was observed in the extent of platelet activation as measured by plasma β-TG between the groups (data not shown).

### Pharmacological inhibition of the PDE3A pathway with cilostazol and CMH development

The plasma cilostazol readings were negligible in mice before the diet introduction (0.15 ± 0.15 ng/mL) and increased in the 0.03% (1175 ± 303.2 ng/mL), 0.3% (3676 ± 495.6 ng/mL), and 3% (4088 ± 670.4 ng/mL) groups. These levels are comparable to the plasma levels of cilostazol in humans following a 200 mg dose [23]. The mean cilostazol plasma levels were similar in the 0.3 and 3% groups and significantly higher than in the control and 0.03% groups. Based on this, a 0.3% cilostazol supplemented diet was used for the pharmacological inhibition studies.

**Fig. 5:**
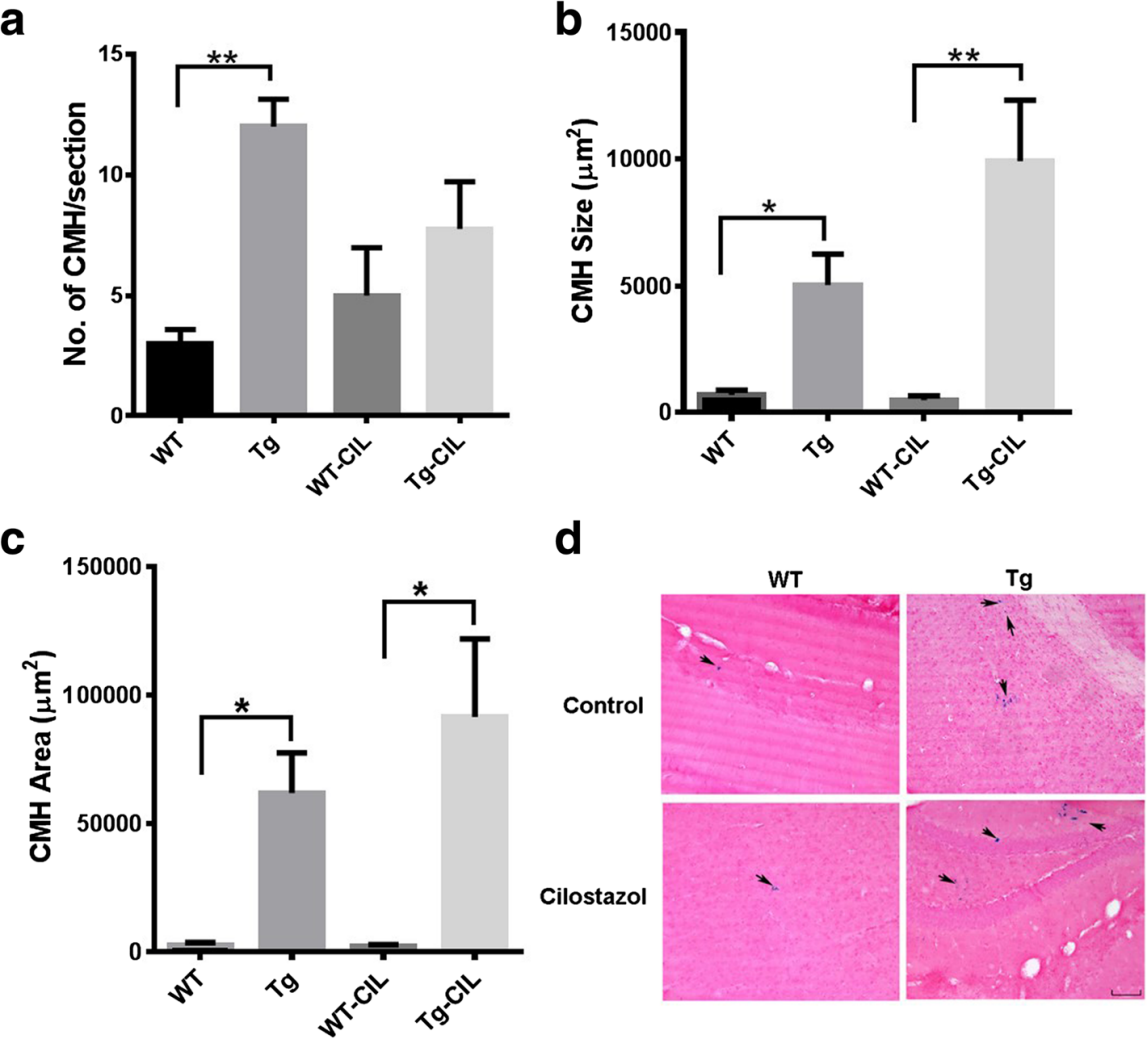
Pharmacological inhibition of PDE3A and CAA-associated spontaneous CMH development. Significant increase in Prussian blue (PB)-positive CMH number (**a**), size (**b**), and area (**c**) in the Tg mice compared with the WT mice. No significant effect of PDE3A inhibition by cilostazol on any of these CMH parameters. Representative images of PB-positive lesions (indicated by *arrows*) in WT and Tg mice with or without cilostazol treatment (**d**). Data are presented as mean ± SEM. Statistical test: one-way ANOVA with Bonferroni’s post-test or Kruskal-Wallis test with Dunn’s post-test. *p < 0.05, **p < 0.01

In the studies examining the effect of cilostazol on CAA-associated spontaneous CMH development, we found that APP/Tg2576 (Tg) mice had a significantly higher number of spontaneously developed CAA-associated PB-positive CMH (12 ± 1.1 CMH per brain section; *p* < 0.01) compared with the WT littermates (3 ± 0.6 CMH per brain section) (Fig. 5a). Ten weeks of treatment with cilostazol, however, did not significantly alter the number of PB-positive CMH in both the Tg (8 ± 2 CMH per brain section) and WT (5 ± 2 CMH per brain section) mice as shown in Fig. 5a. Similarly, the average size and total area of the PB-positive lesions were significantly higher in the Tg mice compared with the WT littermates, and cilostazol treatment did not affect the size and area in WT or Tg mice (Fig. 5b, c). The PB-positive lesion size and total area was 701 ± 189 and 2608 ± 970 μm^2^ in the WT mice and 497 ± 161 and 2086 ± 800 μm^2^ in the WT-CIL mice, respectively. Similarly, the PB-positive lesion size and total area was 5040 ± 1206 and 61,971 ± 15,561 μm^2^ in the Tg mice and 9918 ± 2412 and 91,594 ± 30,473 μm^2^ in the Tg-CIL mice, respectively. Representative images showing PB-positive lesions in the WT and Tg mice with and without cilostazol treatment are shown in Fig. 5d. Overall, Tg mice had significantly higher CAA-associated PB-positive CMH development compared to WT mice, and 10-week cilostazol treatment did not alter PB-positive CMH development in the WT and Tg mice.

**Fig. 6:**
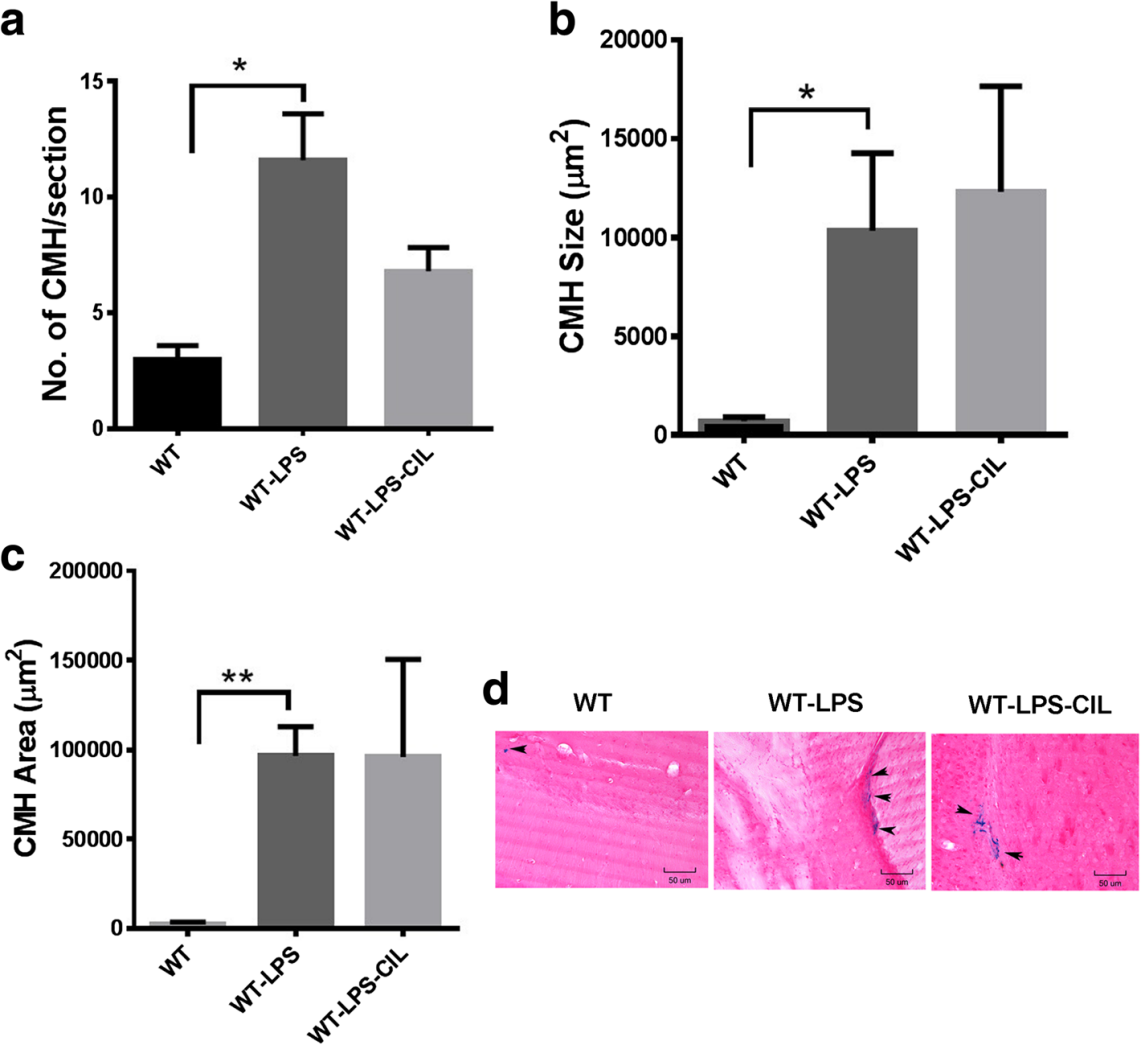
Pharmacological inhibition of PDE3A and inflammation-induced CMH development. Significant increase in LPS-induced PB-positive CMH number (**a**), size (**b**), and area (**c**) in the WT mice compared with PBS controls. Representative images of PB-positive lesions (indicated by *arrows*) in WT controls, mice treated with LPS or with LPS and cilostazol (**d**). No effect of cilostazol treatment on any of these CMH parameters. Data are presented as mean ± SEM. Statistical test: one-way ANOVA with Bonferroni’s post-test or Kruskal-Wallis test with Dunn’s post-test. *p < 0.05, **p < 0.01

In the studies examining the effect of cilostazol on inflammation-induced PB-positive (subacute) CMH development, we found that the PB-positive lesion number, size, and area were significantly higher in the LPS-treated WT mice fed regular diet compared with the PBS controls fed regular diet (Fig. 6a–c). Cilostazol treatment did not alter the PB-positive lesion number, size, or total area in the LPS-treated WT mice (Fig. 6a–c). Representative images showing PB-positive lesions in the WT, WT-LPS, and WT-LPS-CIL mice are shown in Fig. 6d. Overall, 10-week cilostazol treatment did not alter PB-positive inflammation-induced CMH development.

### Genetic and pharmacological modulation of the PDE3A pathway, neuroinflammation, and blood–brain barrier function

**Fig. 7:**
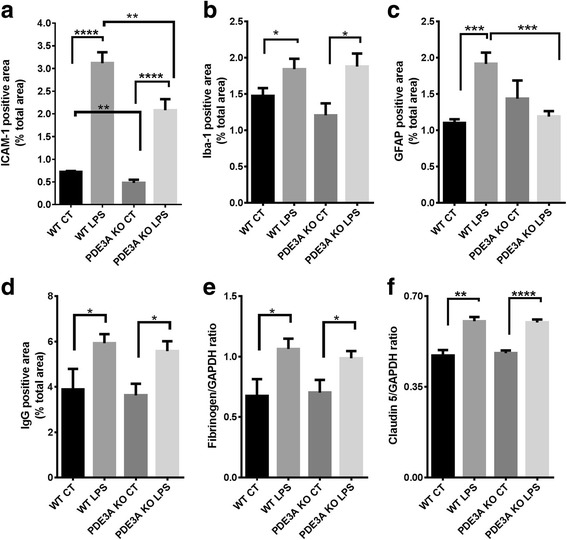
Effect of genetic deletion of PDE3A on neuroinflammation and BBB function in the inflammation-induced CMH mouse model. Significant increase in ICAM-1 (**a**), Iba-1 (**b**), and GFAP (**c**) positive immunoreactive area in the LPS-treated WT and LPS-treated PDE3A KO mice compared with their respective saline controls. Significant reduction in ICAM-1 in both the saline-treated PDE3A KO and LPS-treated PDE3A KO mice compared with their respective WT controls (**a**). Significant reduction in GFAP positive immunoreactive area in the LPS-treated PDE3A KO mice compared with LPS-treated WT mice (**c**). No effect of PDE3A deletion on Iba-1. Significant increase in brain IgG (**d**), fibrinogen (**e**), and claudin-5 (**f**) in the LPS-treated WT and LPS-treated PDE3A KO mice compared with their respective saline controls. No significant difference in any of these markers of BBB function with PDE3A deletion. Data are presented as mean ± SEM. Statistical test: one-way ANOVA with Bonferroni’s post-test or Kruskal-Wallis test with Dunn’s post-test. *p < 0.05, **p < 0.01, ***p < 0.001, ****p < 0.0001

**Fig. 8:**
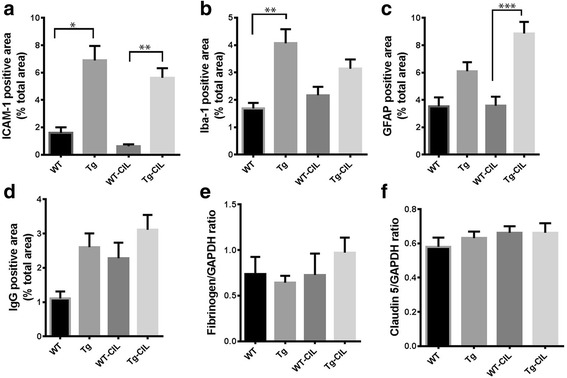
Effect of pharmacological inhibition of PDE3A on neuroinflammation and BBB function in the CAA-associated CMH mouse model. Significant increase in ICAM-1 (**a**) and Iba-1 (**b**) positive immunoreactive area in the Tg mice compared with the WT mice. No effect of PDE3A inhibition with cilostazol on ICAM-1 and Iba-1. Significant increase in GFAP positive immunoreactive area in the Tg mice treated with cilostazol compared with treated WT mice (**c**). No significant difference in brain IgG (**d**), fibrinogen (**e**), and claudin-5 (**f**) between Tg and WT mice. Pharmacological inhibition of PDE3A had no effect on any of these markers of BBB function. Data are presented as mean ± SEM. Statistical test: one-way ANOVA with Bonferroni’s post-test or Kruskal-Wallis test with Dunn’s post-test. *p < 0.05, **p < 0.01, ***p < 0.001

Figures 7 and 8 show the effect of PDE3A modulation on the various markers of neuroinflammation and BBB function in the inflammation (LPS)-induced and CAA-associated CMH mouse models.

In the inflammation-induced CMH mouse model used for the PDE3A KO studies, LPS treatment produced a significant increase in brain levels of ICAM-1 (a marker of endothelial activation) (Fig. 7a), markers of neuroinflammation (Iba-1 and GFAP) (Fig. 7b, c), and BBB function (IgG, fibrinogen, and claudin-5) (Fig. 7d–f) in the WT and PDE3A^−/−^ KO mice compared with saline-treated controls. With genetic deletion of PDE3A, there was a significantly reduced ICAM-1 positive immunoreactive area in both the presence and absence of LPS. GFAP positive immunoreactive area was significantly lower in the LPS-treated PDE3A^−/−^ KO mice compared with the LPS-treated WT littermates (Fig. 7a, c). Genetic deletion of PDE3A did not alter microglial activation (Iba-1) or BBB function (IgG, claudin-5, and fibrinogen), either in the presence or absence of LPS.

In the CAA-associated CMH mouse model used for the pharmacological inhibition studies with cilostazol, the ICAM-1 and Iba-1 positive immunoreactive areas were significantly higher in the Tg mouse brains compared with the WT littermates, while increases in the GFAP positive immunoreactive area in the Tg mice reached statistical significance only in those mice treated with cilostazol (Fig. 8a–c). Markers of BBB function (brain parenchymal IgG, fibrinogen, and claudin-5) did not differ significantly between the WT and Tg mice (Fig. 8d–f). Cilostazol treatment had no significant effect on any marker of endothelial activation, neuroinflammation (except GFAP), or BBB function in either WT or Tg mice compared with their respective regular feed controls (Fig. 8a–f). In the inflammation-induced CMH mouse model, cilostazol treatment did not reduce markers of endothelial, astrocyte, or microglial activation, or injury to BBB (data not shown).

## Discussion

In the current study, we investigated the effects of PDE3A pathway modulation in two mouse models of CMH: the inflammation-induced mouse model and the amyloid precursor protein transgenic mouse model that develops CAA and spontaneous CMH. Our main finding is that PDE3A pathway modulation, either by genetic deletion of PDE3A or pharmacological inhibition of the PDE3A isoform by cilostazol, did not alter CMH development. This effect of PDE3A pathway modulation on CMH development is consistent with no reduction in BBB injury that was observed with both genetic deletion of PDE3A and cilostazol treatment.

CMH is the pathological substrate of CMB and represents a hemorrhage-prone state of the brain. In the current study, we used two models of CMH. Because inflammation plays a central role in CMB development [16], in our first model, LPS was used to induce the formation of CMH. Consistent with previous work, LPS treatment resulted in robust development of acute H&E-positive CMH, and LPS-induced acute CMH formation in this model was associated with an increase in endothelial activation (ICAM-1), markers of neuroinflammation (Iba-1 and GFAP), and BBB injury (IgG and fibrinogen) [16]. Interestingly, LPS-induced acute H&E-positive CMH development was accompanied by an increase in claudin-5 levels. Claudin-5 is a dynamic tight junction protein that is shown to undergo temporal changes with BBB disruption, and studies report increased claudin-5 expression with BBB disruption [24]. This may explain the elevated claudin-5 levels in mice treated with LPS that have increased BBB permeability as indicated by an increase in brain IgG and fibrinogen (Fig. 7d, e). Further, LPS-induced acute CMH were associated with peripheral TNF-α levels, corroborating other work showing a link between peripheral inflammation and CMH development [25]. These results support the notion that inflammation-induced injury at multiple sites of the cerebral vasculature results in reduced BBB function, which is central to acute CMH development [26].

PDE3, also known as cGMP-inhibited PDE, comprises two subfamilies, PDE3A and PDE3B. Both isoforms show unique expression patterns; PDE3A is highly expressed by the cardiovascular system, whereas PDE3B is highly expressed in the cells involved in lipid metabolism and glucose regulation [27]. Both isoforms are expressed in mouse brain regions including the cortex, subcortex, and cerebellum [28]. Inhibition of PDE3A, either by genetic manipulation or pharmacologically, increases the levels of cAMP, thus interfering with platelet activation pathways [29]. PDE3A inhibition also provides anti-oxidant, anti-inflammatory, and microvascular vessel wall protection [3]. These properties of PDE3A inhibition make this approach particularly attractive for CMH treatment when both ischemic and hemorrhagic lesions coexist [3]. PDE3A inhibition by cilostazol has been reported to protect the BBB via mechanisms including nitric oxide production [30], MMP-9 modulation [15, 31], protection of tight junction proteins [11, 32], and increase in phospho-CREB [32]. These effects of cilostazol appear to result in reduced hemorrhagic transformation in mouse models of ischemic stroke [15, 31].

In the current study, genetic deletion of the PDE3A isoform did not alter CMH development in the LPS-induced mouse model of CMH and provided no protection against BBB injury and microglial activation. PDE3A KO mice treated with LPS had lower survival compared with the other experimental groups, for reasons that are unclear. Genetic deletion of PDE3A significantly reduced LPS-induced markers of peripheral (serum TNF-α) and brain (ICAM-1 and GFAP levels) inflammation. Reduction in LPS-induced peripheral TNF-α in the PDE3A KO mice in the current study is consistent with previous work showing significant reduction in LPS-induced TNF-α production in vitro with PDE3A inhibition [33]. Peripheral TNF-α and brain ICAM-1 and GFAP were significantly associated with CMH development in WT mice [16]. However, in spite of reduction in these markers of inflammation, we saw no reduction in LPS-induced CMH development in the PDE3A KO mice. We also observed that the peripheral TNF-α levels were not associated with LPS-induced CMH in the PDE3A KO mice. These results imply that BBB injury and microglial activation, rather than endothelial and astrocyte activation, are involved in LPS-induced CMH development, a subject requiring further investigation.

Clinical evidence shows that the use of platelet medications increases the prevalence of CMB and ICH in patients with ischemic stroke and CAA [3]. In CAA patients, presence of CMB predicts the risk of future ICH, and studies show that the use of anti-thrombotic agents increases the risk of ICH in patients with CAA [34]. A recent study in a transgenic mouse model of CAA reported protective effects of PDE3A inhibition on degeneration of pericytes and microvascular smooth muscle cells, as well as improvement in perivascular drainage and rescue of CAA-associated behavior deficits, when applied early and long term [35]. The same study reported a dramatic increase in PDE3A expression in cerebral vessels severely affected by CAA. In our study, 22-month-old Tg2576 showed robust development of CAA-associated spontaneous PB-positive CMH; these subacute or chronic CMH were primarily associated with markers of neuroinflammation rather than active BBB dysfunction, consistent with previous work [36]. The dose of cilostazol used in our experiment was comparable to that used by Maki and group [35]; however, we did not observe significant changes in CAA-associated number, size, or area of spontaneous CMH after PDE3A inhibition. Further, cilostazol intervention in Tg2576 mice did not affect neuroinflammation and BBB function. The lack of changes in Tg2576 mice may be due to the shorter period of intervention (10 weeks in the current study vs 8, 14, or 17 months, reported in [35]) and the late start of intervention (22 months old), when Tg2576 mice have significant CAA and CAA-associated CMH. Prophylactic intervention may be more efficient than therapeutic treatment (e.g., 10 weeks in the current study), for the modulation of CAA and CAA-associated CMH.

This is the first study to report the effects of genetic deletion of the PDE3A isoform on neuroinflammation and BBB injury associated with microscopic lesions such as CMH. A limitation of our study is that we cannot rule out the possibility that global inhibition of the PDE3A pathway in this model caused an increase in other PDE isoforms that are ubiquitously expressed in the brain [37] as a compensatory mechanism to degrade cAMP [38, 39]. Another limitation of our study is that the work analyzed only inflammation- and CAA-induced CMH, and did not address other causes of CMH, e.g., hypertension. Further, two different histology protocols were used for the genetic deletion and pharmacological inhibition studies: H&E for acute CMH detection in the genetic deletion studies and PB for the detection of subacute CMH in the pharmacological inhibition studies. Finally, absence of protection against CMH development in these models does not rule out a potential therapeutic role for PDE3A modulation in mixed cerebrovascular disease. Given the known anti-thrombotic effects of PDE3A inhibition, absence of enhanced CMH development is modestly encouraging. This positive feature is mitigated by the absence of BBB protection in these studies.

## Conclusions

Our study showed that modulation of the PDE3A pathway does not alter development of CMH. These findings were similar for mouse models using either genetic deletion or pharmacological inhibition of the PDE3A pathway. Neither CMH development nor BBB function was altered with PDE3A pathway modulation in both the inflammation-induced and CAA-associated mouse models of CMH. The role of microglial activation and BBB injury in CMH development warrants further investigation.

## Abbreviations

BBB: Blood–brain barrier
CAA: Cerebral amyloid angiopathy
CMH: Cerebral microhemorrhages
DAB: 3,3′-Diaminobenzidine
H&E: Hematoxylin and eosin
LPS: Lipopolysaccharide
PB: Prussian blue
PDE3A: Phosphodiesterase 3A
